# AGEs accumulation is related to muscle degeneration and vascular calcification in peritoneal dialysis patients

**DOI:** 10.1590/2175-8239-JBN-2020-0119

**Published:** 2021-02-26

**Authors:** Laís de Faria Fonseca, Anna Beatriz Araújo, Kélcia Rosana da Silva Quadros, Cinthia Esbrile Moraes Carbonara, Sérgio San Juan Dertkigil, Andrei Carvalho Sposito, Rodrigo Bueno de Oliveira

**Affiliations:** 1 Universidade Estadual de Campinas, Faculdade de Ciências Médicas, Laboratório para o Estudo do Distúrbio Mineral e Ósseo em Nefrologia, Campinas, SP, Brasil.; 2 Universidade Estadual de Campinas, Faculdade de Ciências Médicas, Departamento de Clínica Médica, Campinas, SP, Brasil.; 3 Universidade Estadual de Campinas, Faculdade de Ciências Médicas, Departamento de Radiologia, Campinas, SP, Brasil.

**Keywords:** Renal Insufficiency, Chronic, Muscle Strength, Uremia, Vascular Calcification, Insuficiência Renal Crônica, Força Muscular, Uremia, Calcificação Vascular

## Abstract

**Background:**

Patients with chronic kidney disease (CKD) are affected by dynapenia, sarcopenia, and vascular calcification. Advanced glycation end products (AGEs) may accumulate in peritoneal dialysis (PD) patients and favor sarcopenia via changes in collagen cross-linking, muscle protein breakdown, and the calcification of arterial smooth muscle cells via p38-MAPK activation. The aim of this study is to explore the relationships between AGEs, muscle degeneration, and coronary artery calcification.

**Methods:**

This was a clinical observational study in patients with CKD undergoing PD, in which serum and skin AGEs (AGEs-sAF), cumulative glucose load, muscle strength and functional tests, muscle ultrasounds with elastography, coronary artery calcium (CAC) quantification, and muscle density by multislice computed tomography were measured.

**Results:**

27 patients aged 48±16 years, dialysis vintage of 27±17 months, had AGEs-sAF levels of 3.09±0.65 AU (elevated in 13 [87%] patients), grip strength levels of 26.2±9.2 kg (11 [42%] patients with dynapenia), gait speed of 1.04±0.3 m/s (abnormal in 14 [58%] patients) and "timed-up-and-go test" (TUG) of 10.5±2.2s (abnormal in 7 [26%] patients). Correlations between AGEs-sAF levels and femoral rectus elastography (R=-0.74; p=0.02), anterior-tibialis elastography (R= -0.68; p=0.04) and CAC (R=0.64; p=0.04) were detected. Cumulative glucose load correlated with femoral rectal elastography (R=-0.6; p=0.02), and serum glycated hemoglobin concentrations correlated with psoas muscle density (R= -0.58; p=0.04) and CAC correlated with psoas muscle density (R=0.57; p=0.01) and lumbar square muscle density (R=-0.63; p=0.005).

**Conclusions:**

The study revealed associations between AGEs accumulation and lower muscle stiffness/density. Associations that linked muscle degeneration parameters with vascular calcification were observed.

## Resumo

**Histórico:**

Pacientes com doença renal crônica (DRC) são afetados pela dinapenia, sarcopenia e calcificação vascular. Produtos finais da glicação avançada (AGEs) podem se acumular em pacientes em diálise peritoneal (DP) e favorecer a sarcopenia por meio de alterações em ligações cruzadas do colágeno, quebra da proteína muscular e calcificação das células do músculo liso arterial por meio da ativação da p38-MAPK. O objetivo deste estudo é explorar as relações entre AGEs, degeneração muscular e calcificação da artéria coronária.

**Métodos:**

Este foi um estudo clínico observacional em pacientes com DRC submetidos à DP, no qual foram medidos os AGEs séricos e teciduais (AGEs-sAF), a carga cumulativa de glicose, a força muscular e testes funcionais, ultrassonografias musculares com elastografia, quantificação do cálcio da artéria coronária (CAC), e a densidade muscular por tomografia computadorizada multislice.

**Resultados:**

27 pacientes com idade entre 48±16 anos, tempo de diálise entre 27±17 meses, tinham níveis de AGEs-sAF de 3,09±0,65 UA (elevado em 13 [87%] pacientes), níveis de força de preensão de 26,2±9,2 kg (11 [42%] pacientes com dinapenia), velocidade de marcha de 1,04±0,3 m/s (anormal em 14 [58%] pacientes) e teste "timed-up-and-go" (TUG) de 10,5±2,2s (anormal em 7 [26%] pacientes). Foram detectadas correlações entre os níveis AGEs-sAF e a elastografia do reto femoral (R=-0,74; p=0,02), a elastografia tibial anterior (R= -0,68; p=0,04) e a CAC (R=0,64; p=0,04). A carga cumulativa de glicose se correlacionou com a elastografia do reto femoral (R=-0,6; p=0,02), as concentrações séricas de hemoglobina glicada se correlacionaram com a densidade muscular do psoas (R= -0,58; p=0,04) e o CAC se correlacionou com a densidade do músculo psoas (R=-0,57; p=0,01) e a densidade do músculo quadrado lombar (R=-0,63; p=0,005).

**Conclusões:**

O estudo revelou associações entre o acúmulo de AGEs e menor rigidez/densidade muscular. Foram observadas associações que ligavam parâmetros de degeneração muscular com a calcificação vascular.

## Introduction

Chronic kidney disease (CKD) is highly prevalent worldwide and is associated with high morbidity and mortality rates as a result of numerous complications^1^^-^ 6.

Recent studies have revealed a high prevalence of sarcopenia in patients with CKD, the presence of which can lead to such unfavorable outcomes as bone fractures, high hospitalization rates and mortality^7^^,^ 8.CKD has been documented to induce a catabolic state mediated by inflammatory mechanisms and metabolic derangements, including malnutrition, insulin/insulin-like growth factor-1 resistance, and pro-inflammatory cytokine expression. Inflammatory processes triggered by reduced renal function and uremic toxins result in imbalances between muscle tissue repair and degradation. One consequence of such imbalance is a reduction of muscle synthesis^9^^-^ 12.

This complex pathophysiology of skeletal muscle degradation in CKD has common features with the mechanisms for cardiovascular disease development, namely the interplay of multiple factors such as oxidative/nitrative stress, inflammation, and uremic toxins^13^. It is speculated that advanced glycation end products (AGEs) are involved in the genesis of vascular calcification and sarcopenia^14^^-^ 18.

AGEs are believed to act through their specific receptor (RAGEs) in muscles and vessels, resulting in inflammation, endothelial dysfunction, vascular calcification, and pathological alteration to the blood flow of skeletal muscles^15^^,^ 16. In addition, muscle proteins such as beta-enolase, actin, and creatine kinase have been observed to target glycation with aging or CKD^17^. Evidence from clinical settings shows that AGEs accumulation in the body is associated with low hand grip strength, slow gait speed, and increased muscle weakness^19^^,^ 20.

CKD patients on peritoneal dialysis (PD) are potentially more likely to form and accumulate AGEs because the reactive carbonic compounds present in the body diffuse into the peritoneal cavity and join with the reactive carbonic compounds in the dialysate^21^. Solutions used for PD have high levels of glucose in their composition; the heat sterilization used for these solutions can cause AGEs and pro-oxidant molecule generation^22^.

The aim of this study was to explore the relationship between the accumulation of AGEs (in skin and serum) and parameters related to skeletal muscle quality, quantity, and function, as well as with coronary artery calcium accumulation in patients with CKD on PD.

## Material and Methods

This was a clinical, observational and cross-sectional pilot study conducted among clinically stable CKD patients on PD at the Nephrology Service of the Hospital de Clínicas of the State University of Campinas (UNICAMP) from June 2018 to April 2019. Written informed consent was obtained from all subjects, and the ethics committee of UNICAMP approved the study protocol under the CAAE number 79826317.8.0000.5404. The study was performed in accordance with the precepts of the Declaration of Helsinki.

During the study inclusion period, 45 patients were in a PD program at the unit. All patients were invited to participate in the study and assessed according to the following inclusion criteria: having stage 5 CKD according to the KDIGO criteria^23^, being in a chronic PD program for more than 3 months, age > 18 years, and able to grant free and informed consent in a form. The exclusion criteria were: the presence of severe and uncontrolled infectious or inflammatory disease, a diagnosis of hematologic or solid organ cancer, chronic liver disease or jaundice, a history of organ transplantation, the presence of amputation, mobility restriction or accident sequelae, and cerebrovascular disease that made walking difficult or impossible.

Clinical, demographic, and laboratory data such as age, gender, body mass index (reference range: 18.5 a 24.9 kg/m^2)^, diagnosis of diabetes mellitus, PD vintage, kt/V, renal function, residual diuresis, and cumulative glucose load were evaluated. Patients were treated either by continuous ambulatory peritoneal dialysis (CAPD) or automated peritoneal dialysis (APD). The dialysate used by patients had glucose concentration ranging from 1.5 to 4.25% and calcium ion concentration from 2.5 to 3.5%. To calculate the cumulative glucose load, daily glucose exposition was estimated from the dialysate glucose concentration in relation to the daily total infused volume and multiplied by 30 and by the number of months on PD treatment, obtaining a value in kilograms of glucose. Laboratory tests were performed using the following methods: hemoglobin - automated (reference range: 10 - 12 g/dL); albumin - colorimetric (bromocresol green) (reference: > 3.5 g/dL); calcium - colorimetric (reference range: 8.8 - 10.2 mg/dL); phosphorus - UV phosphomolibidate (reference: < 5.5 g/dL); glycated hemoglobin - high performance liquid chromatography (HPLC) (reference: < 5.7%), and parathormone (PTH) by electrochemiluminescence (Roche(r), USA) (reference range: 15 - 65 pg/mL).

### Evaluation of calcium accumulation in coronary arteries and muscle density

Non-contrast multislice cardiac computed tomography (CT) was employed to perform coronary artery calcium (CAC) detection and quantification via an electrocardiographically driven volumetric acquisition mode with a tube voltage of 120 kV and collimation width of 3.0 mm (Canon Aquilion CT 64, Canon System, Japan). The images were analyzed using the software dedicated to the CAC score (Vitrea, Calcium Scoring, Vital Images, Japan). The Agatston method was used to express the values of coronary calcification (reference value: = 0)^24^. The densities of lumbar square and psoas muscles (expressed in Hounsfield units) were evaluated.

### Quality, quantity, and function assessment of skeletal muscles

Elastography (Toshiba Aplio 500; 14 MHz high frequency linear transducer) of the following lower limb muscles was performed: femoral rectus, gastrocnemius, and anterior tibialis. This yielded data on muscle thickness (in mm), elastography (muscle stiffness expressed by tissue conduction velocity, in m/s), and ultrasound signal intensity. Functional tests of muscle strength and performance were then conducted, in which handgrip strength (in kg) assessments were performed using a Saehan SH5001 hydraulic dynamometer (Saehan Corporation, Changwon, 51342, Korea). Measurements were taken in the dominant arm, with the patient in sitting position and the elbow at a 90-degree angle. Averages between the results of three measurements were considered for analysis. Values ​​of 30 kg for men and 20 kg for women were considered as the cutoff^25^. Patients with handgrip strengths below these values were diagnosed with dynapenia. Dynapenia was considered as the loss of muscle strength or power regardless of muscle size^8^.

For functional evaluations, "timed-up-and-go" test (TUG) and gait speed test were applied. In the TUG test, the patient was instructed to get up from a chair, walk a 3-m linear path, then return and sit in the original position. A time of 9.2 s was established as a cutoff reference, corresponding to the 25^th^ percentile of the distribution curve of this parameter in the study sample. In the gait speed test, the patient was instructed to travel a timed distance of 10 m at normal pace from which the initial 2 m (acceleration) and final 2 m (deceleration) were disregarded. The mean time between the three measurements was considered as the average gait speed of each patient. The cutoff point was set at the speed of 1 m/s^25^.

### Evaluation of ages accumulation in skin (ages-saf) and serum

The accumulation of AGEs through skin auto-fluorescence (AGEs-sAF) was measured using the AGE-Reader^TM^ device (DiagnOptics BV, Groningen, The Netherlands) according to the manufacturer's recommendations. This device non-invasively measures fluorescence emitted by the skin that is influenced by the intensity of AGEs deposition. The device calculates the relationship between emitted and reflected excitation light. In this study, AGEs-sAF levels were expressed in arbitrary units (UA) and measured in triplicate on the ventral side of the forearm. Areas with arteriovenous fistulas, scars, and tattoos were avoided for clarity of reading. According to the manufacturer, the AGE-Reader^(TM)^ and its software have been validated in patients with a 6-percent photo-type skin reflection index (Fitzpatrick class I to IV). Individuals whom had black skin color (Fitzpatrick classification V and VI) were not measured for AGEs-sAF due to their skin reflectance index <6%, according to manufacturer instructions^26^^,^ 27.

The reference values ​​of the AGEs-sAF levels were grouped by age as follows: 20 to 29 years, 1.53 arbitrary units (AU); 30 to 39 years, 1.73 UA; 40 to 49 years, 1.81 UA; 50 to 59 years, 2.09 UA; 60 to 69 years, 2.46 UA; 70 to 79 years, 2.73 UA; and > 80 years, 2.71 UA^28^. Serum glycated hemoglobin levels were measured as a direct way to quantify circulating AGEs.

### Statistical Analysis

Data were expressed according to the mean ± SD or median and interquartile range (showed in parenthesis), as appropriate. Mean comparisons were performed using a Student's t-test or the Mann-Whitney test for continuous variables. Spearman's rank correlation coefficient was used to evaluate the relationships between AGEs-sAF and selected variables. The threshold for statistical significance was set at p < 0.05. All statistical analyses were performed using SPSS software (version 22.0, SPSS Inc., Chicago-IL).

## Results

Twenty-seven patients, 14 of which were women (52%), aged 48 ± 16 years with dialysis vintages of 27 ± 17 months were included in the study. Arterial hypertension was observed as the main cause of CKD in 8 of the patients (30%), followed by diabetes mellitus in 3 (11%), and undetermined causes in 6 (22%). AGEs-sAF levels were above the estimated age value in most patients (13 [87%]). AGEs-sAF level measurements were not possible to perform in 5 (18.5%) patients due to the V-VI skin photo-type and in 7 (26%) patients due to renal replacement therapy modality changes that took place during the data collection period. Clinical, demographic, and laboratory data of the sample as well as results regarding the parameters related to the accumulation of AGEs, CAC score, characteristics and muscle performance tests are recorded in Table 1.

**Table 1:** Clinical and demographic characteristics, data on general laboratory and AGEs-related parameters, CAC score, and skeletal muscle parameters

Parameters	N = 27
Age (years)	48 ± 16
Gender (female, %)	14 (52)
Ethnicity (Caucasoid; N, %)	15 (56)
Peritoneal dialysis vintage (months)	27 ± 17
Weekly kt/V	2,03 ± 0,51
Body mass index (kg/m2)	26,9 ± 6,3
Hemoglobin (g/dL)	10,9 ± 1,5
Albumin (g/dL)	3,6 ±0,6
Calcium (mg/dL)	8,7 ± 0,6
Phosphate (mg/dL)	5 ± 1,2
Parathyroid hormone (pg/mL)	272 (203 – 396)
Related to the accumulation of AGEs	
AGEs-sAF (UA)*	3,09 ± 0,65
Glycated hemoglobin (%)*	5,4 ± 0,5
Cumulative glucose load (kg)	121,3 (63 a 169)
Related to muscle functional performance*	
Hand grip strength (kg)	26,2 ± 9,2
Gait speed (m/s)	1,04 ± 0,3
Timed-up-and-go test (s)	10,5 ± 2,2
Related to muscle quantity or quality*	
Femoral rectus straight thickness (mm)	13,7 ± 3,4
Femoral rectus US-elastography (m/s)	1,71 ± 0,1
Anterior tibialis thickness (mm)	12,4 ± 1,4
Anterior tibialis US-elastography (m/s)	3,05 ± 0,6
Gastrocnemius thickness (mm)	16,9 ± 2,3
Gastrocnemius US-elastrography (m/s)	2,01 ± 0,4
Psoas density (HU)	28,1 ± 12,7
Lumbar square density (HU)	23,4 ± 16,1
Coronary artery calcium score (Agatston)	35 (0 – 291)

AGEs-sAF: advanced glycation end-products-skin autofluorescence; US: ultrasound; HU: Hounsfield unit.

* N = 14 to 26

Almost half of the patients in the sample were diagnosed as having dynapenia (11, [42%], of which 6 [54.5%] were women). Among the patients with dynapenia, lumbar square muscle density was about half of that observed in patients without dynapenia (15.7 vs. 31 HU; p = 0.04). Patients with dynapenia presented a trend of higher cumulative glucose load (143 [103 to 184] vs. 81 [39 to 128] kg; p = 0.06) compared to those without a dynapenia diagnosis. Gait speed was considered outside of reference range in 14 (58%) patients and correlated with age (R = -0.43; p = 0.03), handgrip strength (R = 0.5; p = 0.01), and mean time to TUG (R = -0.69; p = 0.001). TUG results were above reference values in 7 (26%) patients.

### Correlations between ages accumulation, cumulative glucose load, and skeletal muscles parameters

**Figure 1:**
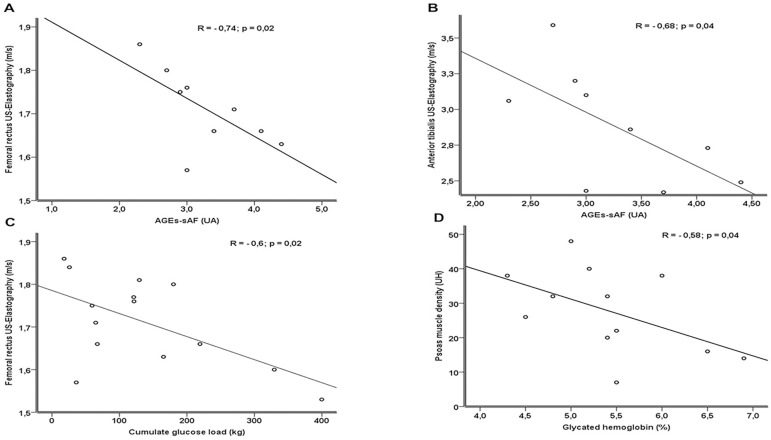
Correlations between advanced glycation end products (AGEs) accumulation in the skin, cumulative glucose load, and skeletal muscle parameters.

AGEs-sAF levels negatively correlated with the elastography of the femoral rectus (R = -0.74; p = 0.02) and anterior tibialis (R = -0.68; p = 0.04). Cumulative glucose loads also negatively correlated with the elastography of the femoral rectus (R = -0.6; p = 0.02). Serum glycated hemoglobin levels negatively correlated with psoas muscle density (R = -0.58; p = 0.04) (Figures 1A to 1D).

### Correlations between skeletal muscle parameters and coronary artery calcium score

**Figure 2:**
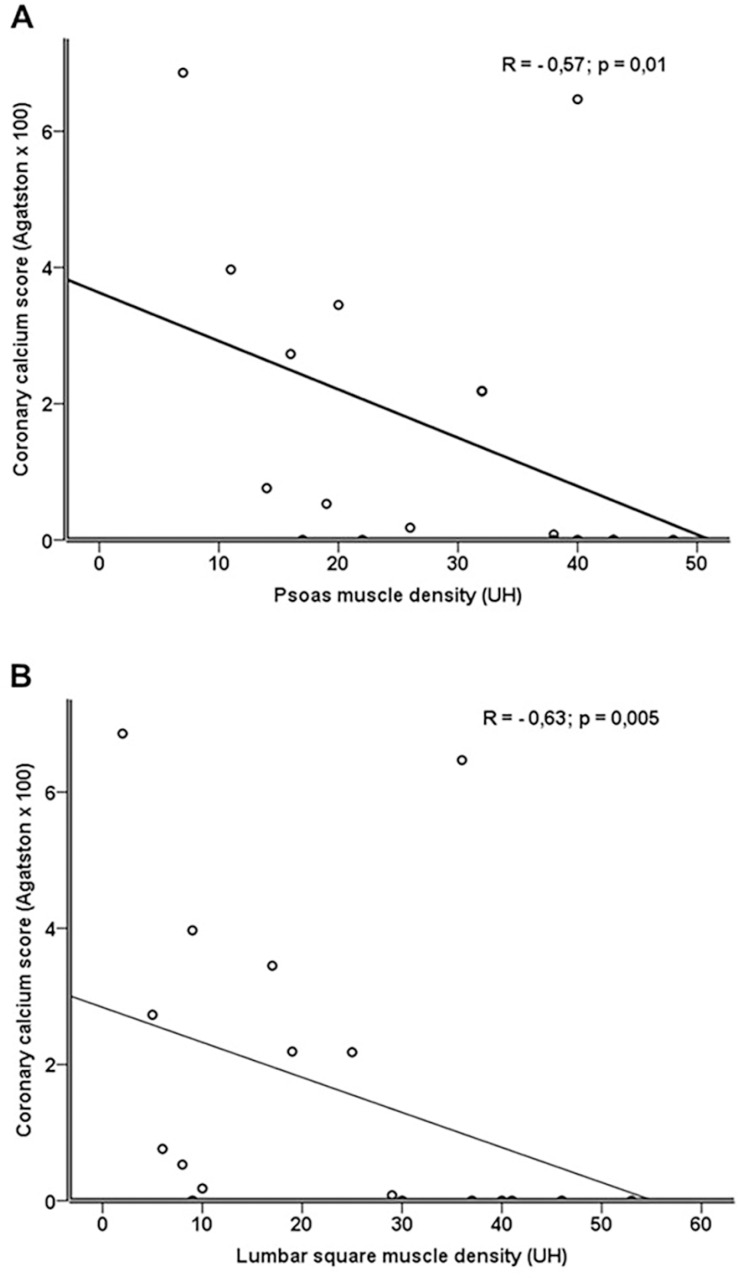
Correlation between muscle density and coronary artery calcium score.

Moderate negative correlations were observed between psoas and lumbar square muscle density and CAC (R = -0.57; p = 0.01 and R = -0.63; p = 0.005, respectively) (Figures 2A and 2B).

### Correlation between ages accumulation in skin and coronary artery calcium score

**Figure 3:**
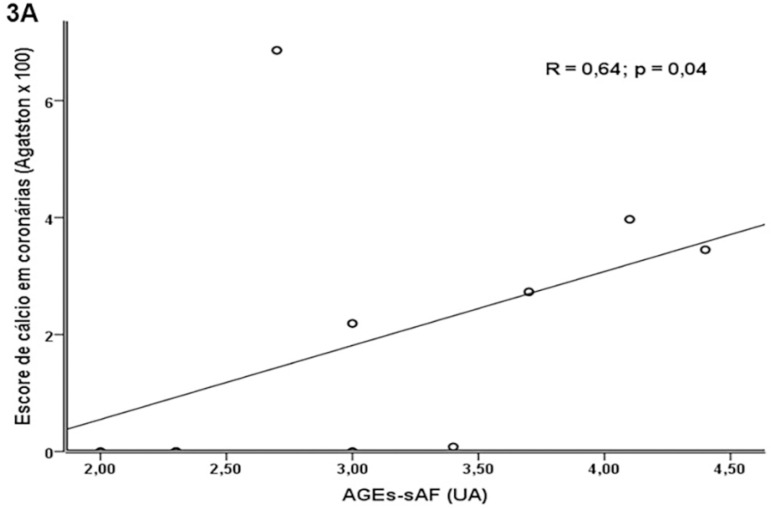
Correlation between advanced glycation end products (AGEs) accumulation in skin and coronary artery calcium score.

Within the sample, 18 patients (67%) underwent CAC score measurements, yielding a median value of 35 (0 - 291) Agatston. Eleven (61%) patients had positive CAC score. A moderate positive correlation was observed between the accumulation of AGEs-sAF and CAC score (R = 0.64; p = 0.04) (Figure 3A).

## Discussion

This study revealed the following main findings: first, elevated levels of AGEs-sAF and abnormal CAC score were detected in most patients. Second, AGEs accumulation was found to correlate negatively with ultrasound elastography and muscle density. Third, muscle density was negatively correlated with CAC score. Finally, AGEs accumulation was found to correlate to both skeletal muscle parameters and CAC score.

Skeletal muscle degeneration in CKD is multifactorial, involving uremic toxins, chronic inflammation, insulin resistance, malnutrition, and oxidative stress^29^. The results of the interaction of these factors with skeletal muscle can be expressed through loss of muscle mass, strength (dynapenia), or functionality. Applying the results of elastographic ultrasound examinations to the structural abnormalities of muscle tissue can be quite complex^30^.

The meaning of ultrasound wave propagation velocity within skeletal muscle has yet to be established fully. The type of muscle, its functional demand, the nature of the lesion and its evolutionary phase may influence the interpretation of elastography results^30^^-^ 33. Clinical data suggests that reduced ultrasound wave propagation velocity in skeletal muscles may indicate lower stiffness, liposubstitution, edema or atrophy, while increased velocity can translate into inflammation and fibrosis^30^.

In the present study, reduced ultrasound wave propagation velocity observed in the femoral rectus and anterior tibialis muscles had a negative correlation with AGEs accumulation, which indicates less muscular stiffness due to atrophy and liposubstitution; these results are compatible with the process of sarcopenia. The same reasoning applies to the interpretation of the negative correlation between muscle density analyzed by CT and the accumulation of AGEs.

Evidence in the literature suggests a relationship between AGEs accumulation and reduced muscle function, dynapenia, or sarcopenia^19^^,^ 34 ^,^ 35, possibly mediated by stress induction and inflammation^29^^,^ 36. However, at present there are no data on the relationship between AGEs accumulation and sarcopenia in patients with CKD on PD.

Clinical studies have documented the relationship between sarcopenia and cardiovascular risk indices, such as carotid intima thickness, epicardial adiposity, and less brachial artery flow-mediated dilation^37^^-^ 39. The accumulation of adipose tissue in skeletal muscle or its liposubstitution produces pro-atherogenic and proinflammatory cytokines with a paracrine effect that promotes coronary disease^40^. This hypothesis is in line with the lower muscle density (as a translation of liposubstitution) and elevated CAC score observed in the current study.

A relationship between AGEs accumulation and the accumulation of calcium in the coronary arteries was also observed. Studies have shown that AGEs affect vascular endothelium and induce foam cell formation, apoptosis, calcium deposition, oxidative stress, and inflammation^15^^,^ 19. These phenomena in combination result in the progression of vascular calcification and atherosclerotic plaque^41^^-^ 44. In turn, the calcification of arteries that supply muscle tissue can impair the muscle regeneration process^15^.

A controversial aspect in the literature is cumulative glucose exposure through peritoneal dialysis solutions and the accumulation of AGEs or potential consequences in muscles. Although our results point to a correlation between cumulative glucose load and skeletal muscle parameters, other authors have reported that hemodialysis patients may have higher accumulation of AGEs than PD patients^45^^,^ 46. Vongsanim et al., observed no clear relationship between biocompatible dialysates and skin auto-fluorescence, suggesting that other factors than PD fluid AGEs content appear more important in determining this parameter^46^.

**Figure 4:**
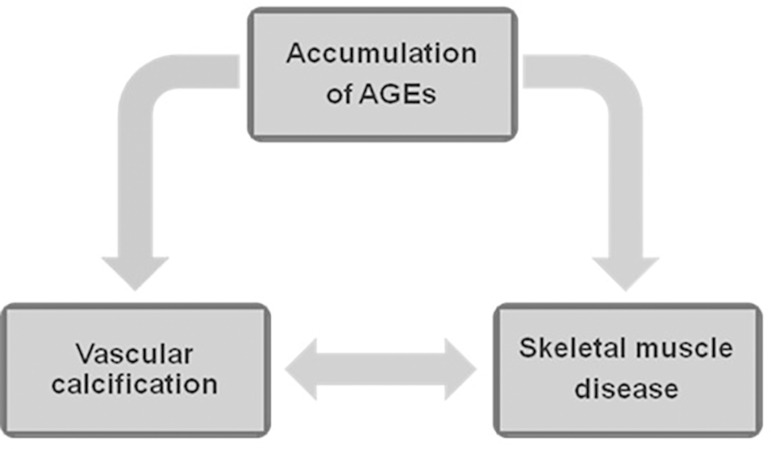
Diagram of the hypothesis about the pathophysiological interaction between muscular disease, vascular calcification, and AGEs accumulation in patients with CKD. AGEs promoted by CKD in addition to excessive glucose exposition from PD solutions contributes to skeletal muscle degeneration. Paracrine changes from the liposubstituted muscle tissue are involved in the development of vascular calcification that in turn impairs the blood supply to skeletal muscles. AGEs: advanced glycation endproducts; CKD: chronic kidney disease; PD: peritoneal dialysis.

The present pilot study had some limitations, such as a sample consisting of a low number of patients from a single center. Further, skeletal muscle biopsies were not performed for specific analysis. Additionally, the methodology used to evaluate skeletal muscle quality by ultrasound with elastography is a new technique and awaits validation in this population. Another limitation of the study is the absence of hemodialysis patients as a comparison group for the analysis. Our findings cannot be extended to all CKD patients since the presence of the uremic environment with all its repercussions could have different impact along CKD stages or in hemodialysis patients. The strength of the study is the hypothesis generated from the results, by which AGEs have a role in the pathophysiology of both skeletal muscle derangements and vascular calcification. According to this hypothesis, there is a reciprocal relationship between muscle disease in CKD and the development of vascular calcification (Figure 4A).

This study reveals associations between AGEs accumulation and lower muscle stiffness/density (likely due to liposubstitution and atrophy) associated with CAC deposition. While interesting, these results are presently inconclusive in terms of the causal relationship between AGEs, sarcopenia, and vascular calcification.

Further studies are needed to address this problem in patients with CKD in PD and to establish whether AGEs-sAF levels, data from ultrasounds with elastography, or skeletal muscle density by CT may serve as surrogate markers of dynapenia or sarcopenia. These surrogate markers could allow early interventions such as dietary counseling, strengthening exercises, and functional m+uscle training to be applied to benefit patients.
